# The metabolic flexibility of quiescent CSC: implications for chemotherapy resistance

**DOI:** 10.1038/s41419-021-04116-6

**Published:** 2021-09-04

**Authors:** Kangchen Chen, Chenzhi Zhang, Sunbin Ling, Rongli Wei, Jianguo Wang, Xiao Xu

**Affiliations:** 1 Department of Hepatobiliary and Pancreatic Surgery, Affiliated Hangzhou First People’s Hospital, Zhejiang University School of Medicine, Hangzhou, China; 2 NHC Key Laboratory of Combined Multi-organ Transplantation, Hangzhou, China; 3 Institute of Organ Transplantation, Zhejiang University, Hangzhou, China; 4 Division of Hepatobiliary and Pancreatic Surgery, Department of Surgery, The First Affiliated Hospital, Zhejiang University School of Medicine, Hangzhou, China

**Keywords:** Cancer metabolism, Cancer microenvironment, Cancer stem cells

## Abstract

Quiescence has been observed in stem cells (SCs), including adult SCs and cancer SCs (CSCs). Conventional chemotherapies mostly target proliferating cancer cells, while the quiescent state favors CSCs escape to chemotherapeutic drugs, leaving risks for tumor recurrence or metastasis. The tumor microenvironment (TME) provides various signals that maintain resident quiescent CSCs, protect them from immune surveillance, and facilitates their recurrence potential. Since the TME has the potential to support and initiate stem cell-like programs in cancer cells, targeting the TME components may prove to be a powerful modality for the treatment of chemotherapy resistance. In addition, an increasing number of studies have discovered that CSCs exhibit the potential of metabolic flexibility when metabolic substrates are limited, and display increased robustness in response to stress. Accompanied by chemotherapy that targets proliferative cancer cells, treatments that modulate CSC quiescence through the regulation of metabolic pathways also show promise. In this review, we focus on the roles of metabolic flexibility and the TME on CSCs quiescence and further discuss potential treatments of targeting CSCs and the TME to limit chemotherapy resistance.

## Facts

- Cancer stem cells (CSCs) are identified in most types of liquid and solid tumors and contribute to tumor onset, chemotherapy resistance, recurrence, and metastasis.
- When the bulk of the tumor cells are eliminated by adjuvant treatments, CSCs may survive in a reversible quiescent state.
- In both adult stem cells and CSCs, low ROS levels are frequently associated with a protective intracellular environment and with the perseverance of stem cell quiescence/dormancy.
- CSCs can reprogram their metabolism to flexibly adapt to environmental changes, which is considered critical for them to enhance the antioxidant compensative capacity and sustain their self-renewal ability.

## Open questions

- What gives rise to the emergence of quiescent CSCs?
- What kind of metabolic flexibility favors the maintenance of quiescent CSCs?
- Which are the therapeutic regimens that quiescent cancer cells might be resistant to and in which manner?
- Does the future lie in combining chemotherapies that target proliferative cancer cells treatment that target quiescent CSCs?

## Introduction

Many of the current chemotherapies are limited to merely targeting proliferative cancer cells. The residual population of chemotherapy-resistant tumor cells capable of regenerating the cancer disease is thought to be enriched in CSCs [1]. CSCs have the principal properties of self-renewal, clonal long-term repopulation potential, and the capability of producing non-stem daughter cells which make up the bulk of tumors [2]. Importantly, CSCs can enter a quiescent state, a reversible cell cycle arrest that is characterized by minimal basal metabolic activity. Recent advances suggest that quiescence is an actively maintained state in which signaling pathways are involved in maintaining a poised state [3]. The entry of the quiescence withstands metabolic stress and preserves its genomic integrity [3, 4]. A recent study tracing glioma stem cells (GSCs) in a transgenic mouse model proved that the quiescent CSCs can survive from temozolomide [5]. In squamous cell carcinoma, TGF-β concentrating near tumor-vasculature bestows slower-cycling properties to neighboring CSCs, which show increased chemoresistance against cisplatin [6]. There is emerging evidence that the ability of CSCs to enter a quiescent state is an important driver of chemoresistance, leaving risks for tumor recurrence. For convenience, we summarize those chemotherapeutic agents which are reported to induce quiescent CSCs (Table 1).

**Table 1:** The chemotherapeutic agents which may cause resistance due to CSCs, and the overview of the pathogenic mechanism of quiescence or enhanced stemness.

Chemotherapeutic agents	Cancer	Overview of the pathogenic mechanism of quiescence or enhanced stemness	Therapeutic regimens or methods to overcome chemoresistance	Reference
Sorafenib	HCC	1. Upon mTORC1 inhibition through sorafenib treatment, elevated *laminin-332* expression was observed to broadly decrease cell mitosis, indicating a *quiescent* state of CSCs; 2. the development of HCC in a microenvironment enriched with ECM proteins, including *laminin-332*, ultimately led to sorafenib-resistant HCC, dependent on the α3β1/Ln-332 axis; 3. the HIF1α/USP22 positive feedback loop in promoting *glycolysis and stemness* on TP53 inactivation, which is known to control the balance between *quiescence* and proliferation in CSCs, in sorafenib-resistance HCC.	1. Ungiven; 2. Ungiven; 3. 2-DG.	[49, 59, 57]
5-fluorouracil	1. Colon cancer 2. HCC	1. *Quiescent CSCs* expressed increased levels of ZEB2 and further upregulated pCRAF/pASK1 levels resulting in increased chemoresistance; 2. 5-FU inhibits CD90^+^ proliferating CSCs, some of which produce CD13^+^ semiquiescent CSCs, while CD13 inhibition suppresses the self-renewing and tumor-initiating ability of *quiescent CSCs*.	1. Ungiven; 2. combining a CD13 inhibitor with a ROS-inducing chemo/radiation therapy	[19, 109]
Cytarabine	Acute myeloid leukemia	1. *Quiescent leukemia stem cells (LSCs)* expressed the highest levels of enhancer of zeste (EZH) 1 and EZH2, the PRC2 catalytic subunits, in the AML hierarchy, and that dual inactivation of EZH1/2 eradicated quiescent LSCs to cure AML; 2. c-MPL-positive cell population within Lin^−^ c-Kit^+^ leukemia cells included a high percentage of *LSCs in a quiescent state*, enhanced colony formation ability, and increased homing efficiency.	1. a novel EZH1/2 dual inhibitor to sensitize LSC to the cytarabine; 2. AMM2, a c‐MPL inhibitor to sensitize LSC to the cytarabine.	[102, 104]
Cisplatin	Breast cancer	**1**. MDA-MB-231PAC10 cells are *quiescent* with a delayed doubling time, which may be caused by the high expression of p21(Waf1).	1. Disulfiram inhibits CSC marker expression and reverses paclitaxel and cisplatin resistance in cells.	[105]
BCR-ABL1 targeted tyrosine kinase inhibitor	CML	1. GLI2 expression enhances leukemic progenitor *dormancy* in stromal co-cultures.	1. SMO inhibition, a clinical antagonist of GLI2, can sensitize LSCs to TKI in vivo at doses manner.	[106]
EGFR TKIs including gefitinb, erlotinib, osimertinib	1. Lung cancer 2. Osteosarcoma cancer 3. Cholangiocarcinoma	1. Culturing on de-cellularized *ECM*, or co-culturing with the *ECM donor cells*, immediately confers resistance to tumor cells that are otherwise sensitive to EGFR TKIs 2. *TGFβ*-miR-499a-SHKBP1 network orchestrates the EMT kinase switch that induces resistance to EGFR inhibitors in CD166^+^ OSCs; 3. In vivo, tumors developed from resistant cholangiocarcinoma cells were larger and exhibited a more prominent stromal compartment, enriched in *cancer-associated fibroblasts (CAF)*.	1. Ungiven. 2. Ungiven. 3. Ungiven.	[56, 65, 67]
Docetaxel	Breast cancer	1. CD10 + GPR77 + *CAFs* are driven by persistent NF-κB activation via p65 phosphorylation and acetylation. CD10 + GPR77 + CAFs promote successful engraftment of patient-derived xenografts, and targeting these CAFs with a neutralizing anti-GPR77 antibody abolishes tumor formation and restores tumor sensitivity.	The neutralizing anti-GPR77	[62]
Oxaliplatin	Colorectal cancer	1. H19 was enriched in *CAF*-derived conditioned medium and exosomes, which in turn promoted the stemness of CSCs and the chemoresistance of CRC cells in vitro and in vivo.	1. Ungiven.	[66]
Gemcitabine	Pancreas cancer	1. Inhibition of *glycolysis* using 2-deoxy-D-glucose (2-DG) significantly enhanced the cytotoxicity of gemcitabine and inhibited the CSC and EMT phenotypes in GR cells both in vitro and in vivo.	1. Ungiven.	[88]

Recent studies have shown that CSCs depend on different metabolic pathways compared to differentiated tumor cells, and the metabolic activities directly participate in the CSC quiescent/proliferative states transition or support tumor progression [7]. CSCs can reprogram their metabolism to flexibly adapt to environmental changes, which is considered critical for them to enhance the antioxidant compensative capacity and sustain their self-renewal ability [8]. Quiescence is a mechanism whereby CSCs can be poised into a low metabolic state. Exploring the role of CSC metabolism and the mechanisms underlying metabolic flexibility has become a major focus in current cancer research. Changes in the environmental supply of metabolic substrates, intrinsic metabolic pathway disturbances by molecular mechanisms, altered reactive oxygen species (ROS) levels, and depolarized mitochondrial membranes of CSCs, may all contribute to quintessential metabolic reprogramming [9].

The CSC quiescence is also associated with the tumor microenvironment (TME), the environment around a tumor, including the surrounding blood vessels, immune cells, fibroblasts, signaling molecules, and the extracellular matrix [10, 11]. The TME components stir the balance of quiescent/proliferative CSCs, preserve their plasticity, and promote CSC stemness, thereby protecting them from immune system attack and resulting in chemotherapy failures [12, 13]. In this review, we describe the roles of quiescent CSC and TME in chemoresistance, depict the metabolic flexibility of quiescent CSC, and further discuss the therapeutic potential of metabolism/TME-based strategies for overcoming chemoresistance.

## Mechanisms of CSC chemoresistance—the multiple lines of self-defense

### Quiescence and chemoresistance

As early as the 1970s, work on the hematologic malignancies predicted that slow-cycling leukemic stem cells cause tumor relapse [14, 15]. Investigators then observed that leukemic stem cells entered into the arrested cell cycle after chemotherapy, much like normal stem cells. The notion that recurrence after standard chemotherapy results from the persistence of quiescent CSCs has been supported recently in several solid tumor types [5, 6, 16]. As discussed below, CSCs trigger a set of complex intracellular molecular and epigenetic programs to enter quiescence, in response to chemotherapies [17].

#### Genetic and epigenetic modifications

Quiescent stem cells are poised for activation by specific energetically favorable mechanisms that are compatible with the low metabolic state of quiescence and that allow for rapid and global responses needed for activation [3]. The Notch, Wnt, and p38-MAPK signaling pathways are the most commonly involved in CSCs quiescence. Kobayashi et al. report that the active p38 mitogen-activated protein kinase 1 (MAPK1) can induce a quiescent state of CSC in prostate cancer [18]. Meanwhile, CSCs can quit the quiescent state under certain circumstances [19]. The Notch signaling and Wnt signaling pathways were reported to regulate adult stem cells division and differentiation, and recently they were proven to promote CSC reawakening [20, 21]. Significantly, c-Myc, as a key element in Wnt canonical pathway, can accelerate the CSC cell cycle progression and promote CSC reawakening, while their inactivation was closely associated with the entry into reversible quiescence [22–25].

Epigenetic modifications consist of heritable changes in gene function without alteration of DNA sequence. Epigenetic modifications include DNA methylation, chromatin remodeling, and noncoding RNAs [26]. As cancers progress, epigenetic modifications regulate transcriptional activation, affecting the entry or exit of CSC into quiescence[27]. In fibroblasts, quiescent cells exhibit tighter chromatin compaction and increases of H4K20me2 and H4K20me3 (demethylation/trimethylation of histone H4 at lysine 20) [28]. Interestingly, Ye et al. documented that SET domain-containing protein 4 (SETD4) epigenetically induced quiescent breast CSCs (BCSCs) by facilitating tighter heterochromatin formation via H4K20me3 catalysis [29]. In melanoma, a small subset of slow-cycling cells with a doubling time of >4 weeks, which showed overexpression of the H3K4 demethylase JAR-ID1B, was reported to correlate with tumor progression and metastasis relapses [30]. In addition, Sharma et al. showed that growth arrest-specific 5 (GAS5), a long non-coding RNA, regulated the quiescent state (arrested cell-cycle) in the CD133+ pancreatic CSC population [31]. Taken together, these genetic and epigenetic modifications act as a switch for regulating quiescence and growth arrest in CSCs, which correlate with aggressive biology and chemoresistance of tumors.

#### Immune escape

Clinical evidence on the existence of the quiescent state of tumor cells came from the transmission of cancer from transplant organ donors to immunosuppressed recipients [32, 33]. Under this condition, the immune system contains but not fully extinguishes cancer cell growth. Such cancer cells in immune escape can give rise to tumor recurrence or metastasis, once meeting permissive TME [34].

How do quiescent CSCs acquire immune tolerance? It has been proved that dormant cancer cells could evade immune surveillance by reducing antigenicity in lymphoma, fibrosarcoma, and T-lymphoma [35–37]. In addition, the expression of the immune checkpoints, such as programmed cell death ligand 1 (PD-L1), can protect cancer cells from T cell killing activity [38]. In addition, the TME can help quiescent cells to escape immune surveillance. Vascular endothelial growth factor A (VEGFA) and angiopoietin-2 (ANGPT2; also known as ANG2) and IL-6 secreted into the TME, concurrently upregulated the expression of the immune checkpoint ligand PD-L1 in tumors [39–41]. Apart from the T cells, natural killer cells may also be fooled by cunning quiescent CSCs. Massague lab showed that upon treatment with the WNT inhibitor DKK1, CSCs are forced into quiescence with the sharp decrease of ULBP (ligands for receptors expressed on NK cells, and NK1.1(+) T cells) and acquire the capability of evasion of NK-cell-mediated attack [42].

### The tumor microenvironment

#### Hypoxic tumor microenvironment

Hypoxia has been identified as a hallmark of cancer [43]. Hypoxia within tumor occurs when the rate of rapidly dividing cancer cells in solid tumors quickly surpasses the rate of neovascularization within tumors. In these nutrient-depleted and oxygen-depleted areas, a hypoxic transcriptional response is orchestrated by hypoxia-inducible factors (HIFs) to make cancer cells adaptive to the hypoxic TME [44]. An increasing number of studies have attempted to unveil the complex but inseparable relationships between hypoxia and CSC phenotypes.

As demonstrated in many studies, a hypoxic environment induces the accumulation of HIF subunits in mesenchymal and cancer cells [45] that bind to hypoxia-responsive elements (HREs) in the promoters of hypoxia target genes [46, 47]. Among these subunits, HIF1α is the most studied and widely appreciated for its functions of supporting neovascularization, preventing cellular differentiation, controlling cellular apoptosis, and activating DNA repairment [48], all of which are associated with chemotherapy resistance. Recently, our laboratory unveiled that ubiquitin-specific protease 22 (USP22) can enhance the stability and transcriptional activity of HIF1α, and HIF1α only promoted USP22 transcription when *TP53* was inactivated. Through the HIF1α/USP22 positive feedback loop of *TP53* inactivation, hypoxic TME promotes stemness features (CD44+ and CD24+) and glycolysis in HCC cells, ultimately resulting in sorafenib resistance [49]. In colorectal cancer cells, CSN8 overexpression induces cell-cycle arrest, upregulates quiescence markers and hypoxia response genes (e.g., GLUT1), and enhances survival against 5-fluorouracil treatment [50]. In addition, hypoxia increases the expression of adenosine receptor 2B (A2BR) in human breast cancer cells through the transcriptional activity of HIF1. The binding of adenosine to A2BR promotes breast CSC (BCSC) enrichment by activating protein kinase C-δ, leading to increased expression of interleukin 6 and NANOG [51]. In addition, HIF1α also plays key roles in promoting CSC phenotypes through ITGA6 forkhead box protein M1 (FOXM1), miR-215, and signal transducer and activator of transcription 3 (STAT3) activation in breast cancer, pancreatic cancer, colon cancer, and glioma, respectively [52–55].

Driven by these mechanisms, a hypoxic TME is frequently associated with a more aggressive tumor phenotype. In addition, understanding the mechanism by which the hypoxic TME affects the quiescence of cancers may provide effective therapeutic opportunities.

#### CSC-specific stroma and quiescent CSCs

##### Extracellular matrix

As an indispensable factor in the TME, the ECM contributes to the induction of CSCs and the initiation of tumors. Interestingly, Wang et al. observed that the resistance to EGFR tyrosine kinase inhibitors (TKIs) was conferred to lung cancer cells that were originally sensitive to TKIs after culturing them on decellularized ECM or coculturing them with ECM donor cells [56]. Azzariti et al. also reported that the development of HCC in a microenvironment enriched with ECM proteins, including laminin-332, ultimately led to sorafenib-resistant HCC, dependent on the α3β1/Ln-332 axis [57]. Among the many components of the ECM that are putatively regarded as initiating factors of chemoresistance, laminin has recently received special attention.

Laminin is a glycoprotein ECM component of the connective tissue basement membrane that promotes cell adhesion. Rohn et al. isolated rat hepatic stellate cells and then seeded them onto uncoated polystyrene (PS) or PS coated with either laminin-521 (PS/LN-521) or laminin-211 (PS/LN-211). PS/LN-521 improved hepatic stellate cells adhesion and better-preserved retinoid stores, as well as quiescence-associated and stem cell-associated phenotypes than PS alone [58]. Moreover, laminin-332 was also observed in the ECM surrounding hepatic CSC-like cells which exhibited a low proliferation rate. Upon mTORC1 inhibition through sorafenib treatment, elevated laminin-332 expression was observed to broadly decrease CSCs mitosis, indicating a quiescent state in CSCs [59]. Taken together, the above results demonstrate that the quiescent state of CSCs is closely linked to the ECM and its components.

##### Cancer-associated fibroblast

Given the important role of the ECM on cancer stemness, cancer-associated fibroblasts (CAFs), as the primary source of ECM production in tumors, are also worthy of our comprehensive discussion. CAFs affect tumor progression and resistance via many mechanisms, including morbid secretion of collagens, fibronectins, and ECM-degrading proteases, production of angiogenic factors, and various proinflammatory cytokines and chemokines [60, 61]. Here, we will emphasize the role of CAFs in promoting cancer stemness.

Su et al. showed that co-injection of CD10^+^GPR77^+^CAFs and breast cancer cells effectively improved the engraftment formation in patient-derived xenograft models. Most importantly, the CAF subset specifically defined by CD10 and GPR77 expression was observed to be correlated with chemoresistance and poor survival in multiple cohorts of breast and lung cancer patients [62]. Liu et al. concluded that CAF-induced lysine demethylase 1 (LSD1, a histone-modifying enzyme) activation in hepatic CSCs can enable their self-renewal ability in HCC. The authors inoculated a mixture of liver CSCs (Cherry^+^-GFP^+^) and primary CAFs into NOD/SCID mice, and as expected, CAFs enhanced the oncogenicity of CSCs by activating Notch3-LSD1 signaling in vivo [63]. Similar carcinogenic effects of CAFs have been reported in colorectal cancer, cholangiocarcinoma, oral squamous cell carcinoma, and osteosarcoma cancer [64–67].

#### Matrix stiffness

Tissue formation and development originating from stem cells are orchestrated by a complex network of both chemical and physical properties, but recently researchers have started to focus on the effects of matrix stiffness on CSC and chemoresistance.

Matrix stiffness (rigidity of extracellular matrix) is mainly depending on the composition and organization of ECM [68]. Huang’s laboratory proved that cancer cells effectively form spheroid-like morphologic shapes resembling stem-like cells in 90 Pa fibrinogen gels (the stiffness of most mammalian tissues ranges from approximately 100 to 3000 Pa), while their growth was retarded in 450 Pa and almost entirely halted in 1050 Pa [10]. In addition, Liu et al. showed that the CSC dormancy induced by 450 and 1050 Pa was initiated by the translocation from the cytoplasm to the nucleus of Cdc42, a regulatory protein capable of mechanotransduction [69]. Meanwhile, Shin et al. confirmed the pathological correlation of matrix stiffness and drug sensitivity against standard chemotherapies of myeloid leukemias in vivo, such as everolimus [70].

**Fig. 1:**
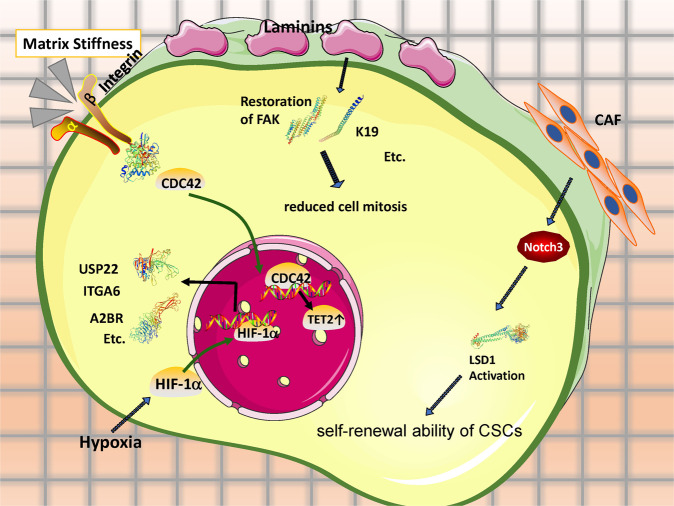
Quiescent CSCs are responsible for refractoriness to chemotherapies via crosstalk of chemical and mechanical signals from TME components, including hypoxia, ECM, CAFs, and matrix stiffness. Through the utilization of antioxidants and available metabolic substrates, CSCs “equips” themselves with metabolic flexibility to maintain themselves quiescent in response to stress and different metabolic austerity. Crucially, inhibition of the morbidly-activated metabolic pathways on which quiescent CSCs are dependent, show promises on chemotherapy sensitization.

Traditional views of the TME solely based on cell–cell or cell–ECM interactions may not thoroughly explain the induction, selection, or preferential maintenance of CSC stemness. Thus, gaining a comprehensive understanding of the interplay between CSCs and their microenvironments may be essential for advancing CSC research and applications (summarized in Fig. 1).

### Other mechanisms of chemoresistance

Other defense lines of CSCs include avoiding cellular and molecular exposure to the drugs, avoiding conditions needed for the drugs to act, damage repair, anti-apoptosis, and regeneration [71]. But their relationships with cancer quiescence remain unveiled, so they are not discussed further in this article.

## Metabolic flexibility

CSCs can reprogram their metabolism to flexibly adapt to environmental changes, which is considered crucial for them to enhance the antioxidant compensative capacity and sustain their self-renewal ability [8, 72]. For example, activation of the glycolytic program in CSCs can enhance their antioxidative capacity, where the pentose phosphate pathway (PPP) is the most relevant and produces reduced intermediates, such as NADPH [72, 73].

Proliferative CSC needs massive biosynthetic materials and an oxidation state to remain growing, while quiescent CSC retains a reduction state for prevention from cell death and injury. It was documented by Anderson et al. that ovarian CSCs were highly flexible/plastic in metabolic phenotypes [74]. Ovarian CSCs were able to accelerate the rate of glycolysis to overcome the ATP inhibition by oligomycin treatment, but conversely, they could also increase the oxygen consumption rate to maintain the proton motive force [74]. Indeed, the metabolic flexibility is not confined to mutual shift between glycolysis and OXPHOS, but also between glycolysis and glutamine metabolism. In colorectal cancer, the metformin-sensitive HT29 cell line showed higher OXPHOS levels, while SW620 cells were metformin-resistant and had lower OXPHOS levels. When glutamine was removed from the culture medium, SW620 cells surprisingly became sensitive to metformin, with decreased expression of stemness biomarkers [75].

**Fig. 2:**
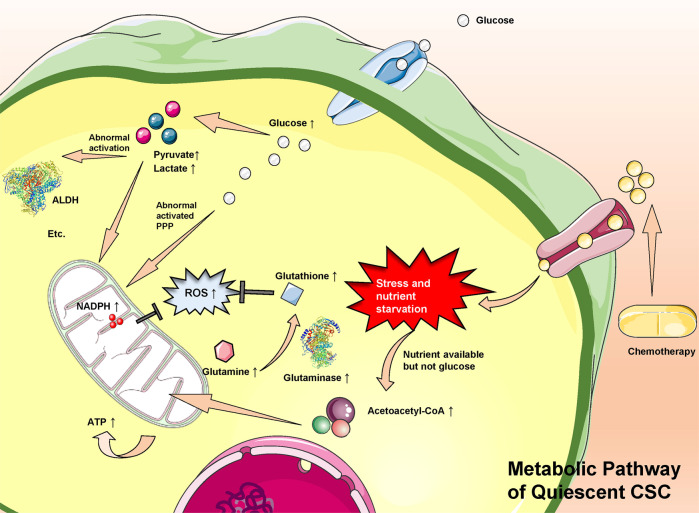
Chemotherapy drugs are taken up by cancer cells, causing stress and nutrient starvation. Through the pentose phosphate pathway, glucose can indirectly produce NADPH, which confronts excessive intracellular ROS to maintain CSC in the quiescent state. Second, when nutrients in the TME are in short, nutrients excluding glucose are decomposed and go through OXPHOS to effectively produce ATP to sustain basic biological demand. Third, glutathione also can be synthesized from glutamine (an important and rich non-essential amino acid) and participate in the self-defense mechanism in response to excessive ROS and reduce its related adverse outcomes. Meanwhile, the intermediates in glycolysis, pyruvate, and lactate further can abnormally activate ALDH, initiating or strengthening CSC stemness.

Crucially, quiescence is a mechanism by which CSCs can be maintained in a low metabolic state, and such a low metabolic state of quiescence is always accompanied by enhanced antioxidant defenses. Oxidative stress was observed to trigger the transition from ROS-low quiescent mesenchymal-like BCSCs (M-BCSCs) to ROS-high proliferative epithelial-like ones (E-BCSCs) [76]. Moreover, increasing evidence demonstrates that glutamine (Gln), as the substrate of reduced glutathione (GSH), also plays a key role in the antioxidant system and serves as an energy source for CSCs [77]. Taken together, lower levels of ROS or enhanced GSH are closely related to the quiescent states of CSC, and even chemoresistance [78, 79]. Reviews of primary metabolic pathways of various CSCs (summarized in Fig. 2) may help to identify quiescent CSCs genes and pathways that maintain the quiescent stem cell state, rendering those cells poised for activation.

### CSC quiescence: balance of excessive ROS and glycolysis

Many studies have revealed that CSCs are prone to exhibiting a glycolytic phenotype compared with their descendants. Indeed, aerobic or anaerobic glycolysis contributes to maintaining CSC phenotypes under specific conditions, such as hypoxia and nutrient limitation [80–82]. As we mentioned above, the quiescent M-BCSCs exhibit higher glycolytic rates in glucose-rich culturing conditions [76]. Theoretically, activation of the glycolytic program can enhance antioxidative capacity, with the PPP being the most relevant and capable of rapidly providing NADPH to meet the massive biosynthetic demand of GSH, which counteracts excessive ROS [83]. Indeed, the quiescent state of CSC is reported to be closely related to lower levels of ROS or enhanced GSH in various cancers [76, 84, 85].

As for the impact of glycolysis on CSC quiescence, glycolysis is considered crucial for CSCs to sustain their antioxidant compensative capacity, enhance stemness, and improve self-renewal ability [9, 49, 76, 86–92]. As shown in Table 2, abnormally increased levels of glycolytic intermediates or products from glycolysis, such as lactate, serine/glycine, and glutamine, have been identified as markers of enhanced CSC stemness and chemotherapy resistance. Given that CSC quiescence ties up with drug sensitivity, it is reasonable to presume that abnormal glycolysis of CSC possibly functions as an initiating factor of chemoresistance. Those metabolic enzymes that initiate the metabolic flexibility to glycolysis are regarded as potential targets to inhibit CSC stemness.

**Table 2:** CSC phenotypes that depend on glycolysis to maintain cancer stemness and the abnormally increased metabolites or metabolic pathway products as markers of enhanced CSC stemness and chemotherapy resistance.

CSC phenotype	Abnormally increased metabolic intermediates as markers of enhanced CSC stemness and chemotherapy resistance	Impacts of glycolysis on CSC stemness or chemotherapy resistance	Impacts of glycolysis on the quiescence/proliferation states of CSCs	Reference
Hepatocellular carcinoma	Lactate and glycolytic ability	The HIF1α/USP22 positive feedback loop upon TP53 inactivation strongly correlates with the CSC subpopulation	The HIF1α/USP22 positive feedback loop in promoting glycolysis and stemness on TP53 inactivation, which is known to control the balance between quiescence and proliferation in CSCs	[49]
Nasopharyngeal carcinoma	ROS and mitochondrial membrane potential	Glycolysis can sustain self-renewal, deter differentiation and enhance the antioxidant system in CSCs	Ungiven	[9]
Breast cancer	ROS	Co-inhibition of glycolysis and thioredoxin and GSH pathways suppresses tumor growth, tumor-initiating potential.	Metabolic or oxidative stress generated by 2DG, H_2_O_2_, or hypoxia promotes the transition of quiescent(ROS^lo^) M-BCSCs to a proliferative (ROS^hi^) E-state.	[76]
Glioblastoma multiforme	Lactate	GBM, particularly the stem cell subpopulation, is sensitive to glycolytic inhibition via lactate dehydrogenase-A inhibitors	Dichloroacetate (100 μM), a compound capable of inhibiting glycolysis metabolism, is capable of hindering CSC proliferation (cell cycle arrest in G2/M phase)	[86]
Glioblastoma	Serine/glycine	Serine/glycine, as intermediates of glycolysis, participates in and promotes the synthesis of purine and thymidine, which are both precursors of RNA and DNA and induce progression of cell division	Hypoxia affects cancer cells in multiple intertwined ways: including a metabolic adaptation with overexpression of all glycolytic pathway enzymes for pyruvate/lactate synthesis and cell growth arrest coexisting with EMT.	[87]
Pancreatic cancer	ROS	Inhibition of glycolysis using 2-DG significantly enhances the cytotoxicity of gemcitabine and inhibits CSC and EMT phenotypes both in vitro and in vivo	Inhibition of glycolysis forces CSCs into the proliferative state and improves chemoresistance against gemcitabine.	[88]
Breast and prostate tumors	Glutamine and glutamate	using secreted frizzled-related protein 4 to inhibit glycolysis is sufficient to inhibit CSC survival in vivo.	Inhibition of glycolysis via sFRP4 makes CSCs vulnerable under conditions of variable glucose content.	[89]
Hepatocellular carcinoma	Mannose 6-phosphate, myo-Inositol-3-phosphate, fructose 6-phosphate, and glucose 6-phosphate	Increased activation of the pentose phosphate pathway diverts glycolytic intermediates to provide precursors for nucleotide synthesis	Ungiven	[90]
Pancreatic cancer	Lactate	Hepatocyte growth factor/c-MET/YAP/HIF-1α signaling enhances the expression of hexokinase 2 (HK2) and promotes glycolytic metabolism	HGF/c-MET/YAP/HIF-1α signaling enhanced the expression of Hexokinase 2 (HK2) and promoted glycolytic metabolism, which may facilitate CSC relatively quiescent state.	[91]
Breast cancer	Unknown	2-DG significantly inhibits the migration and invasion of Hs578Ts(i) and significantly decreases their ability to resist anoikis	Hs578Ts(i)8 showed an increased glycolysis preference and had a significantly increased proportion of cells with relatively quiescent CSC.	[92]

### Oxidative phosphorylation “addiction”

Although many studies have reported that CSCs tend to shift from OXPHOS to glycolysis when facing a nutritional or oxygen supply shortage, OXPHOS is also reported to equip certain CSCs with increased survival from metabolic austerity [81]. OXPHOS can be the primary source of energy and biosynthesis in specific cases as well [74, 93–96]. Here, we review five articles that propose OXPHOS to be the dominant energy resource to maintain CSCs’ self-renewal and tumourigenesis (summarized in Table 3).

**Table 3:** CSC phenotypes that depend on OXPHOS to maintain cancer stemness and abnormally increased metabolites or metabolic pathway products as markers of CSC stemness promotion.

CSC phenotype	Abnormally increased metabolic intermediates as markers of CSC stemness promotion	Impacts of OXPHOS on CSC stemness	Impacts of OXPHOS on the quiescence/proliferation states of CSCs	Reference
Glioma	ATP	Inhibition of glycolysis has minimal effects on energy production in GSCs and progenitor cells. Compared with differentiated cells, GSCs show a higher mitochondrial reserve capacity	GSCs show less glycolytic and rely mainly on OXPHOS than proliferating cells	[93]
Glioblastoma	Unknown	Depletion of IMP2 in gliomasphere, which can depress the oxygen consumption rate and both complex I and complex IV activity, causes impaired clonogenicity in vitro and tumourigenicity in vivo	Inhibition of OXPHOS but not of glycolysis abolishes clonogenicity in slowly-proliferating primary glioblastoma sphere (gliomaspheres), an established in vitro model for CSC	[94]
Lung cancer	Mitochondrial deoxynucleotide triphosphate	The mitochondrial deoxyguanosine kinase is required for the biogenesis of respiratory complex I and mitochondrial OXPHOS, which in turn regulates CSC self-renewal through AMPK-YAP1 signaling	Genetic targeting of DGUOK using doxycycline-inducible CRISPR/Cas9 is able to inhibit OXPHOS activity and lung CSC proliferation	[95]
Pancreatic cancer	Unknown	The MYC/PGC-1a ratio determines the metabolic phenotype of CSCs	Inhibition of mitochondrial complex I exerted by metformin-induced apoptosis preferentially in CSC-enriched cultures while provoking its quiescence	[96]

Vlashi et al. first observed that GSCs and their progenitor cells are less glycolytic than differentiated glioma cells. Their laboratory previously reported that GSCs have lower 26S proteasome activity than nontumorigenic cells [97], and they took advantage of this feature to monitor GSCs in real-time using the fluorescent protein ZsGreen. The GSCs were observed to consume less glucose and produce less lactate while maintaining higher ATP levels than their differentiated progeny [93]. A similar story in gliomaspheres and lung CSCs soon followed this study by Janiszewska et al. [94] and Lin et al. [95]. In addition, Sancho et al. also demonstrated that while pancreatic non-CSCs are heavily dependent on glycolytic substrates, pancreatic CSCs strictly depend on OXPHOS to sustain their vitality. When the CSCs were confronted with mitochondrial respiration inhibition (e.g., metformin administration), they rapidly underwent an energy crisis and apoptosis induced by inhibition of MYC [96].

Indeed, OXPHOS acts as a far more efficient source of ATP production than glycolysis. These OXPHOS-dependent CSCs make efficient use of specific limited nutrients, allowing them to obtain a selective advantage in certain TMEs.

### Potential role of glutamine in CSCs

Overall, most CSC primarily relies on either glycolysis or OXPHOS [98]. However, with the development of tracer techniques, increasing evidence demonstrates that Gln is also an important metabolic substrate and energy source for CSCs [99]. Here, we review articles with results indicating that glutamine and/or glutamate play a significant role in maintaining the stemness of CSCs.

Liao et al. introduced L-asparaginase, an enzyme that catalyses the conversion of glutamine to glutamate, into the culture medium of human non-small-cell lung carcinoma (NSCLC)and pancreatic cancer cells to mimic the effect of decreasing glutamine. Mechanistically, glutamine exhaustion results in an enhancement of intracellular ROS levels through attenuation of the cellular levels of reduced GSH (a derivative of glutamine), ultimately leading to a decreased proportion of CSCs in the tumor in vivo [99]. In addition, there were two indirect lines of evidence. First, knockdown of glutaminase (GLS) 1 significantly suppressed the expression of stemness-related genes, such as CD13 and CD133, and inhibited CSC pool expansion in vitro and tumorigenicity in vivo [100]. Second, a similar result was that GLS1 functioned in accordance with ALDH to maintain cancer stemness in head and neck squamous cell carcinoma [101]. The authors did not deplete or augment the concentration of glutamine in the culture medium in vitro or tissue in vivo; however, given that the function of GLS to hydrolyze glutamine to glutamate was well acknowledged, the role of glutamine in the maintenance of stemness was not negligible.

## ‘Waking up’ quiescent CSCs to overcome chemoresistance

### Therapies targeting CSCs

Resident quiescent CSCs made the prognosis of patients treacherous after chemotherapeutic treatment [102–112]. Even though preventing the activation of quiescent cells has been successful in experimental models [113–116], keeping CSCs long-term dormant may not be feasible in patients. Direct therapeutic elimination of quiescent CSCs awaits a better understanding of their vulnerabilities. Another strategy of overcoming chemoresistance consists of ‘waking up’ this cell population into a differentiated state, making them susceptive to chemotherapies.

#### All-trans retinoic acid

The idea of ‘waking up’ CSCs into a susceptive state to therapies arose from the observation that chemo-resistant leukemic cells became susceptive when they were induced from an undifferentiated state into a differentiated one by the use of all-trans retinoic acid (ATRA) [117]. The success of ATRA therapy inspired other therapies that were based on inducing CSC differentiation in other leukemic malignancies (reviewed by Stahl et al. [118]). However, it was first studied in solid tumors with a well-explored mechanism by Moro et al. [111]. Pretreatment with ATRA, which causes CSCs to differentiate, counteracts cisplatin resistance originating from quiescent NSCLC CD133^+^/CXCR4^+^ cells both in vivo and in vitro. ATRA alone slightly decreases the percentage of CD133+ cells without affecting tumor growth, further demonstrating that ATRA only sensitizes CSCs rather than killing them directly.

#### 2-Deoxy-D-glucose

As mentioned above, quiescence is a mechanism whereby CSCs can be poised into a low metabolic state, therefore, interference with intracellular metabolism shows good practical value and application prospect. 2-deoxy-D-glucose (2-DG) is a glucose molecule that has the 2-hydroxyl group replaced by hydrogen, and it interferes with d-glucose metabolism. There is an increasing focus on using 2-DG to ameliorate resistance to cytotoxic therapies. For example, in triple-negative breast cancer, the more aggressive Hs578Ts(i)8 variant with a significantly increased proportion of CSC phenotype showed an enhanced ability to resist anoikis than its parental cells [92]. Furthermore, Hs578Ts(i)8 exhibited a significantly increased glycolysis flux rather than mitochondrial OXPHOS. After 2-DG was introduced, Hs578Ts(i)8 significantly decreased its ability to resist anoikis. A similar story was reported in Gemcitabine-resistant (GR) pancreatic CSCs. The cytotoxicity of gemcitabine towards GR cells was significantly enhanced when combined with the 2‐DG, manifested by the inhibition of the CSC stemness and the EMT phenotypes both in vitro and in vivo [88].

Previous studies have proved that CSCs possess relatively low intracellular ROS levels, especially in those quiescent ones. To be more specific, the increase of ROS by glycolysis disruption may lead to the differentiation into non-CSCs and the loss of stemness markers [119–121]. Therefore, disrupting ROS equilibrium within quiescent CSCs by 2-DG may be a potential adjuvant to reverse chemoresistance.

#### Oligomycin and rotenone

Oligomycin and rotenone are both mitochondrial OXPHOS Complex inhibitors. Gale et al. reported that combining ATP synthase inhibitor oligomycin A with trastuzumab led to regression of trastuzumab-resistant breast HER2+ tumors in vivo [122]. Matassa et al. also demonstrated that in ovarian cancer, TRAP1 silencing induced resistance to cisplatin, and chemoresistant cells showed over-activated OXPHOS compared with the sensitive counterpart. More strikingly, cisplatin resistance was reversible upon inhibition by metformin/oligomycin [123]. In doxorubicin(DOX)-resistant breast cancer cells, mitochondrial accumulation of DOX in tumor cells was increased by treatment with oligomycin, that is, chemoresistance to DOX was partially reversed at least.

Besides oligomycin A, rotenone also drew some focuses. Cruz-Bermúdez et al. demonstrated that metabolic flexibility from glycolysis to OXPHOS was responsible for cisplatin resistance in NSCLC, and strikingly, the chemoresistance could be reversed by OXPHOS inhibition using metformin or rotenone [124].

#### Other drugs sensitizing CSCs to chemotherapies

Yang et al. used disulfiram (DSF), an inhibitor of ALDH enzyme activity, to induce quiescent-dominated CSCs into proliferative-dominated states and enhance the cytotoxic effect of cisplatin in breast cancer [125]. Similar tactics have been manifested by Jamieson’s laboratory using PF-04449913, also named glasdegib, to sensitize blast crisis LSCs to TKI in vivo at doses that do not affect normal hematopoietic stem cells [106]. Helgason’s laboratory observed that lys05, a highly potent lysosomotropic agent, could promote autophagy inhibition, reverse leukemic stem cell quiescence and drive myeloid cell expansion [107].

### Therapies targeting CSC-specific stroma

Since the TME has the potential to support and initiate stem cell-like programs in cancer cells, targeting the TME components may prove to be a powerful modality for the prevention of chemotherapy resistance. CAFs remodel the tumor ECM and architecture of the TME, leading to poor infiltration of traditional chemotherapies and increased drug resistance. There is an innovative method of preventing chemoresistance by forcing activated CAFs back into quiescence. Sherman et al. documented that vitamin D receptor was over-activated in pancreatic CAFs that could drive tumorigenesis. Importantly, reversion to the quiescent state of CAFs using calcipotriol (a vitamin D analog) witnessed induced stromal remodeling, increased intratumoral gemcitabine infiltration, reduced pancreatic tumor volume, and a 57% sharp increase in survival compared to chemotherapy alone [126]. Aside from calcipotriol, similar findings were reported with ATRA as well. In 3D models and genetic mouse models of PDAC, the use of ATRC to restore the quiescence of CAFs in TME increased vascularity within tumors, improving response to gemcitabine and reducing tumor growth [127].

Considering the presence of CSCs after traditional chemotherapy, treatments for CAFs may have better outcomes when they are combined with traditional chemotherapy.

## Conclusion and perspectives

**Fig. 3:**
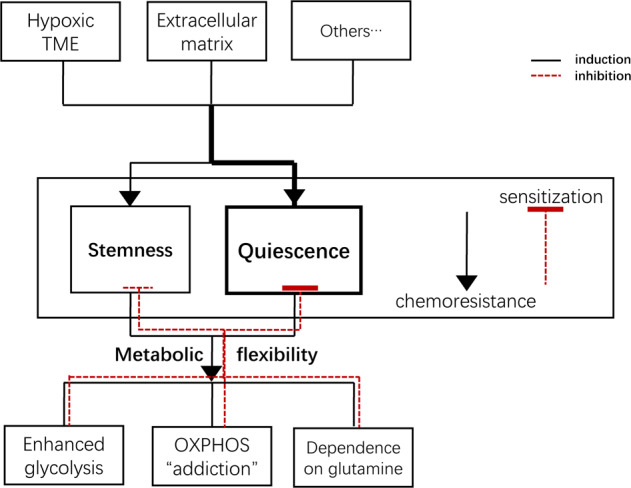
Flow diagram of quiescent CSCs initiation and their metabolic reprogramming. Quiescent CSCs are induced and maintained by different TME components, including a hypoxic tumor environment, laminins, and matrix stiffness of the ECM and CAFs. Quiescent CSCs can reprogram their metabolism to flexibly adapt to environmental changes, which is considered crucial for them to enhance the antioxidant compensative capacity and induce chemotherapy resistance. And it is a promising strategy of making them susceptive to chemotherapies through inhibition of their metabolic flexibility.

There is now solid evidence to support the hypothesis that quiescent CSCs give rise to the refractoriness of chemotherapies in many cancer types. New insights into CSC biology suggest that strategies merely inhibiting CSC stemness characteristics might not suffice to counteract tumor recurrence. The TME maintains the principle properties of CSCs, protects them from immune surveillance, and facilitates their relapse potential. The TME does not only provide various signals that maintain resident quiescent CSCs but also instructs progenitor cells to revert into a stem cell state when the originals are lost [128]. Thus, targeting the TME components may be a more effective strategy for the treatment of chemoresistance than inhibiting the CSCs stemness directly. In addition, through the utilization of antioxidants and metabolic fuels, CSCs “equips” themselves with the metabolic flexibility to maintain themselves quiescent in response to stress and different metabolic austerity [74]. Significantly, primary metabolic pathways of various CSCs are reprogrammed for maintenance in CSCs quiescence, the poised state, but also can be utilized as an effective target of eliminating quiescent CSCs and attenuating resistance to chemotherapies (Fig. 3).

Recently, via molecular imaging methods, such as F^-18^-fluoro-2-deoxy-D-glucose (F^-18^-FDG) positron emission tomography, magnetic resonance imaging, and optical imaging based on the fluorescent protein and principal properties of CSCs, the peritumoral microenvironment can be monitored in real-time and serve as a reflection of the metabolic phenotype of CSCs [97]. With the development of such techniques and methodologies in metabolic research, the measurement of metabolites in the TME can be used to unveil the metabolic phenotype and quiescent/proliferative state of CSCs, providing timely warning of potential chemoresistance and suggestions for the application of anti-tumor treatments.
